# Study of Various Disseminated Intravascular Coagulation Scores and Sequential Organ Failure Assessment Score in Medical Intensive Care Unit

**DOI:** 10.7759/cureus.67134

**Published:** 2024-08-18

**Authors:** Madhulika L Mahashabde, Jugal Sriram

**Affiliations:** 1 General Medicine, Dr. D. Y. Patil Medical College, Hospital and Research Centre, Dr. D. Y. Patil Vidyapeeth, Pune, IND

**Keywords:** sofa score, isth score, poor prognosis, dic score, sepsis

## Abstract

Aim

The aim of the present study was to assess the disseminated intravascular coagulation (DIC) and its correlation with DIC scores (International Society on Thrombosis and Haemostasis (ISTH), sepsis-induced coagulopathy (SIC)) and Sequential Organ Failure Assessment (SOFA) score in medical intensive care unit (MICU) patients.

Methods

The study was conducted at the medical intensive care unit at Dr. D.Y. Patil Medical College and Hospital, D.Y. Patil Vidyapeeth, Pimpri, Pune spanning from October 2020 to September 2022. A total of 100 patients admitted to the hospital ICU satisfying qSOFA score were included in the current study. Approval was obtained from the institutional ethics committee before commencing the study.

All patients and their family members included in the study were provided with a detailed explanation of the study. Clinical history of illness and physical examination were done in detail. The laboratory values were obtained and were calculated with the International Society on Thrombosis and Haemostasis (ISTH), sepsis-induced coagulopathy (SIC) and Sequential Organ Failure Assessment (SOFA) scores.

Results

The average age of the study population was 52.08 ± 16.44 years. Within the study population, 65% were male and 35% were female. Within the group being studied, the average pulse rate was 66.64 ± 17.33 beats per minute, the average systolic blood pressure was 83.7 ± 11.38 mm Hg, the average diastolic blood pressure was 59.7 ± 10.49 mm Hg, and the average respiratory rate was 38.4 ± 4.8. The average Glasgow Coma Scale (GCS) among the participants was 9.51 ± 1.74. The average qSOFA score across the study participants was 2.58 ± 0.6. The study population consisted of 60% survivors and 40% non-survivors. Regarding the study population, 57.15% of individuals experienced mortality as a result of DIC. The statistical analysis revealed a significant difference in the mean ISTH score between the result groups at 48 hours. The disparity in the average SOFA score at admission, 24 hours, 48 hours, day 7 and day 14 between the outcomes (survivors and non-survivors) was statistically significant.

Conclusion

This research suggests that there is a positive link between higher scores on the estimated ISTH, SIC and SOFA scales. The prognosis of critically sick patients is negatively correlated with the progressive increase in DIC scores throughout follow-up, while a stable or declining DIC score is indicative of a more favorable prognosis. There was no significant link seen between non-overt disseminated intravascular coagulation (DIC) mortality and DIC scores.

## Introduction

The disseminated intravascular coagulation (DIC) scoring system is used to diagnose and assess the severity of DIC. International Society on Thrombosis and Haemostasis (ISTH) and sepsis-induced coagulopathy (SIC) [1] scores are widely used for diagnosing DIC, particularly in the context of sepsis, whereas the Sequential Organ Failure Assessment (SOFA) score is a critical tool in prognostication of organ dysfunction [2]. Using platelet count, prothrombin time (PT), fibrinogen and fibrinogen markers ISTH is calculated. ISTH score of 5 or more than 5 indicates DIC. It determines overt DIC and predicts mortality [3]. SIC score using platelet count, SOFA score and PT ratio identifies coagulopathy [4]. A total SIC score of more than 4 indicates a high probability of sepsis-induced coagulopathy among patients diagnosed with sepsis. Using six systems (respiratory, cardiovascular, hepatic, coagulation, renal, neurological) SOFA score assesses organ dysfunction [5]. Each organ system in SOFA score is assigned a score of 0 to 4, with higher scores indicating greater dysfunction. Due to the complexity of the existing DIC scoring systems and the lack of familiarity among clinicians, Iba et al. have recently released guidelines for diagnosing sepsis-induced DIC that may be easily implemented at the bedside [6]. For septic patients with low platelet count, it is recommended to use a two-step process for diagnosing sepsis-induced coagulopathy. This process involves assessing the prothrombin time (PT)/International Normalized Ratio (INR) and platelet levels, as well as calculating the SOFA score [7]. If the SOFA score and PT/INR indicate sepsis-induced coagulopathy, the next step is to use the ISTH overt-DIC score.

There is currently no universally accepted benchmark score to definitively determine the presence of DIC and establish its diagnosis in patients with septic shock. This lack of consensus has sometimes resulted in DIC being humorously referred to as "Disseminated International Confusion." Critical care clinicians have the option to utilize many grading systems, all of which have inherent flaws. Initially, the existing scoring systems are unable to detect the early stages of asymptomatic DIC, which are also referred to as "pre-DIC" or "non-overt" stages. During these stages, the coagulation system is activated but compensated. However, patients in these stages are just as severe as those diagnosed with overt DIC upon admission to the intensive care unit (ICU), and they have the same mortality rate [8].

Disseminated intravascular coagulation (DIC) arises from the extensive and continuous activation of blood clotting, resulting in the accumulation of fibrin in blood vessels or small blood vessels. This process hampers the sufficient blood flow to different organs [9]. Various factors may create a hemostatic imbalance, leading to a condition of hypercoagulability. Inflammatory cytokines play a crucial role in causing this imbalance. There is a definite connection between coagulation and inflammatory systems, where inflammation triggers the activation of the clotting cascade, and the resulting coagulation further enhances inflammatory activity [10]. The hematologic derangements seen in DIC are mainly caused by four distinct mechanisms: heightened thrombin production, inhibition of anticoagulant pathways, compromised fibrinolysis, and activation of inflammation [1].

Upon exposure to detrimental stimuli such as infection and trauma, the body exhibits the secretion of pro-inflammatory cytokines, including interleukin 6, interleukin 1, TNFα, and gamma interferon. These cytokines stimulate the liver to secrete acute-phase proteins, including C-reactive protein (CRP) and fibrinogen. Elevated levels of fibrinogen are seen during the early stages of sepsis. The erythrocyte sedimentation rate (ESR) is a measure of how quickly red blood cells (RBC) settle down in a vertical tube due to their rouleaux production [11]. It indirectly reflects the quantity of fibrinogen present in the blood. Higher levels of fibrinogen may lead to an elevated ESR. Typically, ESR levels rise within 24 to 48 hours. Elevated ESR may result from acute inflammation caused by viral illnesses, tissue damage, ischemia or tumors [12]. Endothelial dysfunction is a crucial factor in the development of sepsis and is accountable for the hematological alterations that take place during sepsis [13]. Endothelial injury often arises from the introduction of bacterial endotoxins or the impact of pro-inflammatory cytokines. This may subsequently result in microvascular coagulopathy and acute organ damage. Heparin influences this process through its anticoagulant property thus playing a crucial role in mitigating coagulopathy and protecting against subsequent organ damage [14].

The present study aims to assess the disseminated intravascular coagulation and its correlation with DIC scores (International Society on Thrombosis and Haemostasis (ISTH), sepsis-induced coagulopathy (SIC) and Sequential Organ Failure Assessment (SOFA) score) in MICU patients.

## Materials and methods

The study was conducted at the medical intensive care unit at Dr. D.Y. Patil Medical College and Hospital, D.Y. Patil Vidyapeeth, Pimpri, Pune spanning from October 2020 to September 2022. A total of 100 patients admitted to medical ICU satisfying qSOFA score were included in the current study. Approval was obtained from the institutional ethics committee before commencing the study.

Inclusion criteria: All patients admitted to the medical intensive care unit and all patients with sepsis (satisfying qSOFA criteria) including diabetes mellitus.

Exclusion criteria: Patients and their family members who were not willing and patients who were immunocompromised.

Methodology

Patients admitted to the medical ICU who satisfied the qSOFA score were selected for the study. The clinical criteria for the qSOFA score include altered mental status, respiratory rate more than or equal to 22 and systolic blood pressure less than 100. A positive qSOFA score of 2 or more than 2 suggests a high risk of poor outcomes in patients with suspected infection. Patients meeting qSOFA score criteria should have infection considered even if it was not considered previously [15].

Each patient and their family members were explained about the study in detail and informed consent was taken.

A specific proforma is used for the collection of data containing complete hemogram, renal function test, liver function test, ESR, CRP, fibrin degradation products, Pt-INR and activated partial thromboplastin time (APTT), serum electrolytes, arterial blood gas (ABG) with lactate, plasma glucose levels, urine routine microscopy, blood and urine cultures, chest X-ray, ECG and 2D Echo.

**Figure 1:**
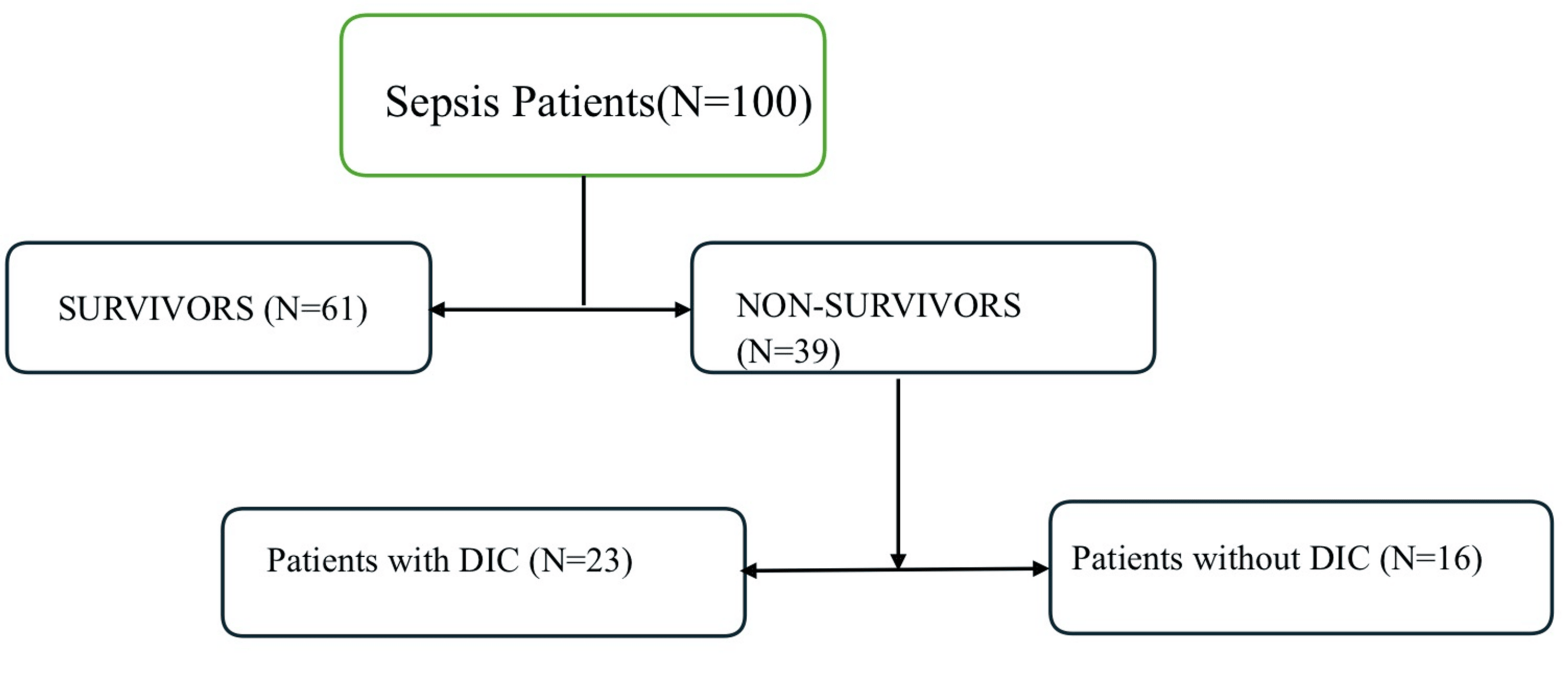
Flowchart showing the outline of the study DIC: Disseminated intravascular coagulation

The observation of 100 patients was recorded and a master chart was prepared. The laboratory values were obtained and were calculated with ISTH, SIC and SOFA scores (Figure 1).

In addition to the initial laboratory investigations defined earlier, the current study also involves hemodynamic and systemic examination of patients along with the Glasgow Coma Scale. Special laboratory investigations like quantitative fibrinogen and D-dimer assays were performed along with routine investigations.

Various clinical parameters like length of ICU stay, necessity of mechanical ventilation, inotrope support, and renal replacement therapy were monitored. The final outcome of each patient was evaluated, considering whether they survived or not. Additionally, DIC and SOFA scores were assessed on the day of admission and serially every 48 hours until the patient was discharged. These scores are indicators of organ dysfunction and the prognosis of the patient.

Patients were regularly followed up with regular clinical assessment, review of medical records and laboratory investigations for 28 days or four weeks at the medical ICU to monitor various lab parameters of DIC scores. At that period patients were grouped into survivors and non-survivors after which 28 days mortality rate was determined. The study carefully analyses how patients developed DIC and how this affected their risk of death. The main goal was to see how patients died within 28 days and to determine if the scores used to assess their condition were linked to their survival changes. The secondary outcomes like changes in scores, score parameters and cause of death help in providing a comprehensive evaluation of patients' condition and factors that influence mortality and morbidity over a period of time.

Statistical methods

Statistical Package for Social Science (SPSS) was used to analyze the observed data. All the numerical data of the study was expressed as ranges and mean ± SD. Categorical data was expressed as percentage.

P value <0.05 was considered statistically significant. The number of cases at the time of study divided by all participants examined gave a prevalence rate. Pearson’s correlation coefficient was used to understand the relationship between the parameters of the study. The cutoff points used were <0.3 for weak correlation, 0.3 to 0.7 for moderate correlation and >0.7 for strong correlation. To test the significance of qualitative data, a Chi-square test was used.

## Results

The average age of the study population was 52.08 ± 16.44. Within the study population, 65% were male and 35% were female. In the group being studied, the average pulse rate was 66.64 ± 17.33, the average systolic blood pressure (SBP) was 83.7 ± 11.38, the average diastolic blood pressure (DBP) was 59.7 ± 10.49, and the average respiratory rate was 38.4 ± 4.8. The average Glasgow Coma Scale (GCS) score in the study sample was 9.51 ± 1.74. The average qSOFA score across the study population was 2.58 ± 0.6. The study population consisted of 60% survivors and 40% non-survivors. Regarding the study population, 57.15% of individuals experienced mortality associated with DIC. Patient details are mentioned in Table 1 below.

**Table 1:** Patient details SBP: systolic blood pressure; DBP: diastolic blood pressure; GCS: Glasgow Coma Scale; DIC: disseminated intravascular coagulation; qSOFA: Quick Sequential Organ Failure Assessment

Parameter	Mean ± SD	Median	Minimum	Maximum
Age	52.08 ± 16.44	48.52	17.00	93.00
Sex	Frequency		Percentages	
Female	35		35	
Male	65		65	
Vitals	Mean ± SD	Median	Minimum	Maximum
Pulse	66.64 ± 17.33	64.00	42.00	138.00
SBP	83.7 ± 11.38	82.00	62.00	112.00
DBP	59.7 ± 10.49	62.00	42.00	94.00
Respiratory Rate	38.4 ± 4.8	39.00	29.00	49.00
GCS	9.51 ± 1.74	8.00	7.00	15.00
qSOFA	2.58 ± 0.6	4.00	3.00	4.00
Outcome	Frequency		Percentages	
Survivors	61		61	
Non-survivors	39		39	
Death	Frequency		Percentages	
Death due to DIC	23		58.9	
Death due to other causes (non-DIC)	16		41	

The research population did not show a statistically significant difference in the mean ISTH score on admission and the mean ISTH score on day 14 between different outcomes. The p-value is more than 0.05. The statistical analysis revealed a significant difference in the mean ISTH score between the result groups at 48 hours (Table 2). The p-value is less than 0.05.

**Table 2:** Comparison of ISTH score between survivors and non-survivors ISTH: International Society on Thrombosis and Haemostasis

Parameter	Outcome (Mean ± SD)		P-value
	Survivors (N=61)	Non-survivors (N=39)	
ISTH SCORE ON ADMISSION	3.94 ± 0.86	3.98 ± 1.22	0.892
ISTH SCORE 24 Hrs	4 ± 1.26	4.92 ± 1.8	0.001
ISTH SCORE 48 Hrs	3.48 ± 2.04	6.14 ± 1.46	<0.001
ISTH DAY 7	4.10 ± 0.18	5.81 ± 0.87	0.032
ISTH SCORE ON DAY 14	4.33 ± 0.341	6.21 ± 0.22	0.780
ISTH SCORE ON DAY 28	4.64 ± 0.642	7.683 ± 0.32	0.007

The research population did not show a statistically significant difference in the mean SOFA score on day 14 across different outcomes (Table 3).

**Table 3:** Comparison of SOFA score between survivors and non-survivors SOFA: sequential organ failure assessment

Parameter	Outcome (Mean ± SD)		P-value
	Survivors (N=61)	Non-survivors (N=39)	
SOFA SCORE ON ADMISSION	11.33 ± 2.68	12.76 ± 2.64	<0.001
SOFA SCORE 24 Hrs	11.4 ± 4	14.16 ± 2.68	<0.001
SOFA SCORE 48 Hrs	10.5 ± 2.012	15.03 ± 3.0	<0.001
SOFA SCORE day 7	10.0 ± 2.5	13.9 ± 3.58	<0.001
SOFA SCORE DAY 14	9.58 ± 2.02	13.52 ± 3.0	0.316
SOFA SCORE DAY 28	9.002 ± 2.0	13.1 ± 3.02	<0.001

The research population did not show a statistically significant difference in the mean SOFA score on day 14 across different outcomes. The disparity in the average SOFA score at admission, 24 hours, 48 hours, day 7 and day 14 between the outcomes was statistically significant (Table 4).

**Table 4:** Comparison of SIC score between survivors and non-survivors SIC: Sepsis-induced coagulopathy

Parameter	Outcome (Mean ± SD)		P-value
	Survivors (N=61)	Non-survivors (N=39)	
SIC SCORE AT ADMISSION	4.06 ± 1.14	5.16 ± 1.12	<0.001
SIC SCORE 24 Hrs	3.87 ± 1.18	5.19 ± 1.08	<0.001
SIC SCORE 48 Hrs	4.06 ± 1.1	5.08 ± 3.87	<0.001
SIC 7	3.02 ± 1.1	4.62 ± 1.39	<0.001
SIC DAY 14	3.51 ± 0.5	5.0 ± 0.751	<0.001
SIC DAY 28	3.81 ± 0.61	5.41 ± 0.71	0.355

## Discussion

The use of the abbreviation 'DIC' to represent 'death is coming' serves as a reminder that there is still much progress to be achieved in the treatment of this very frequent disease. DIC is not a standalone pathology but rather a secondary condition that arises as a result of the advancement of other diseases. It is believed to affect around 1% of patients who are admitted to the hospital due to sepsis [16]. The presence of DIC is usually subordinate to some underlying disease. The morbidity and fatality rates are influenced by both the primary illness and the extent of coagulopathy [17]. Patients with DIC, i.e. with higher scores, had higher mortality rates when compared to non-DIC patients [16].

In 2010, Singh et al. [18] performed a retrospective cohort analysis on critically sick patients with DIC. The research focused on consecutively hospitalized adult patients who were 18 years old or older. It was observed that the incidence rate of DIC per 100,000 person-years reduced from 26.2 in 2004 to 18.6 in 2010. Except for the age range of 18 to 39 years, the frequency of DIC (disseminated intravascular coagulation) rose with age in both males and females, but continuously remained greater in males [18]. In our study, there is a higher incidence in men, while in Singh et al.'s study, a decreasing trend was observed over six years of study. Our study found no changes in trend over a period of two years.

In the present investigation, the DIC score at admission was shown to be greater in patients who experienced mortality, as opposed to those who either lived or showed improvement. However, this difference did not reach statistical significance. Nevertheless, those who died with DIC had a notably higher DIC score compared to those who died from other reasons or improved with no statistical significance [19]. Our research found a strong link between the DIC score, SIC score, and SOFA score with the outcome of patients. These values were measured on day 0, day 1, day 2, day 7, day 14, and day 28. Within our research, the study population consisted of 61% survivors and 39% non-survivors. The SURPRISE research, conducted by Ros et al. in 2018, used a prospective, observational cohort design to evaluate the prognosis of acutely hospitalized critically sick patients [20]. In the research, individuals who were projected to die within a year were found to have a lower number of comorbidities, such as chronic obstructive pulmonary disease (COPD) and diabetes, compared to other patients. The average Acute Physiology Age and Chronic Health Evaluation (APACHE) APACHE III score for patients with a projected survival of over a year was 59.6, but for those with a predicted death within a year, the average score was 82.0. The research found that the average accuracy for predicting 1-year survival was 81%, and the kappa value was 0.43 [20].

In a study conducted by Rostom et al. [21], the aim was to investigate the correlation between DIC and increased mortality, the impact of changes in ISTH scores of DIC patients, the prognostic significance of DIC and SOFA scores in critically ill patients, and the predictive ability of these scores. The results showed that 96.8% of individuals who did not survive had lower DIC scores upon admission compared to their scores at the time of death. On admission, DIC score values were greater than or equivalent to the levels before discharge in 94.4% of non-survivors. The composite ratings of DIC and SOFA showed a substantial correlation with both mortality and the ultimate outcome inside the intensive care unit. When our study is compared with the study conducted by Rostom et al., both studies aim to evaluate and compare the prognostic values of DIC scores in predicting patient outcome. Iba et al. [22] performed research to identify a valuable indicator of anticoagulant treatment in patients suffering from sepsis and diffuse intravascular coagulation (DIC). The study revealed that the predictive accuracy of SOFA was much greater (80.5%) compared to overt-DIC (66.7%, P .001). The mortality rate was significantly reduced in those with an improved SOFA score compared to those without improvement [23]. Although our study includes a diverse range of treatments and different demography of patients it gives us a direct comparison to Iba et al.’s study while considering the same outcome.

The Sequential Organ Failure Assessment (SOFA) demonstrated the most robust association with 28-day mortality in individuals with sepsis and disseminated intravascular coagulation (DIC). The study employed a scoring system established by the International Society of Thrombosis and Haemostasis (ISTH) to diagnose and predict the severity of disseminated intravascular coagulopathy (DIC) [19]. This system demonstrated excellent sensitivity and specificity by considering various criteria such as the existence of an underlying disorder, platelet count, prothrombin time, quantitative D-dimer, and fibrinogen levels. A score of five or above shows the presence of overt DIC, while a score below five does not definitively exclude DIC but may suggest the presence of non-overt DIC. Consistent with our findings, Battah et al. did research in 2010 which showed that the DIC score during the first 48 hours was a reliable indicator of the clinical progression and prognosis [24]. However, the second research only focused on patients with sepsis and used SOFA and DIC scores exclusively during the first 48 hours of hospitalization. In addition, they only used two of the necessary coagulation variables, namely platelet count and prothrombin time, in order to construct the DIC score. Our study encompassed a range of causes for admission to the intensive care unit (ICU). We utilized the ISTH, SIC, and SOFA scores at specific time points (day 0, day 1, day 2, day 7, day 14, and day 28) throughout the patient's stay in the ICU, as well as at the time of discharge or death. Additionally, we incorporated all four essential parameters required to calculate the ISTH score for disseminated intravascular coagulation (DIC), which include platelet count, prothrombin time, quantitative serum fibrinogen level, and D-dimer level.

The study underscores the importance of using multiple scoring systems (ISTH, SIC and SOFA) for prior diagnosis of DIC and timely intervention. Understanding the further advancement of these scores can help clinicians predict the patient outcome and tailor the treatment accordingly. The study signifies the relation between DIC scores and patient mortality confirming the prognostic values of the scoring system thus answering the aim of the study. Using the established scoring systems and correlating the scores are the strengths of the study.

The study’s limitations are the quantity and diversity of data. Patient heterogeneity is another limitation as patients have a wide range of underlying conditions and might be at different stages of illness progression. The study being a single-center study may limit the generalizability of the results. Patient confounding factors such as comorbid conditions and different timelines of intervention could influence the outcome of the study.

## Conclusions

This research suggests that there is a positive link between higher scores on the computed (ISTH, SIC and SOFA) scales with both illness severity and death rate. The prognosis of critically sick patients is negatively correlated with the increasing trend of DIC scores throughout follow-up, while a declining or stable trend of DIC scores is linked with a better prognosis. There was no significant link seen between non-disseminated intravascular coagulation (DIC) mortality and DIC scores. The integration of several scoring models (SOFA, ISTH, SIC) significantly enhances and greatly augments the predictive capability of any individual model. Our research showed that DIC scores might serve as a potentially valuable indicator for assessing DIC in critically sick patients upon admission to the ICU, as well as for predicting their unfavorable outcomes at an early stage.
